# System-Specific Metabolomic Clocks Reveal Aging Heterogeneity and Links to Diet, Cognition, Mortality

**DOI:** 10.1093/geroni/igaf122.4204

**Published:** 2025-12-31

**Authors:** Anastasia Leshchyk, Nicole Roth, Stefano Monti, Stacy Andersen, Thomas Perls, Paola Sebastiani

**Affiliations:** Tufts Medical Center, Boston, Massachusetts, United States; Boston University, Boston, Massachusetts, United States; Boston University, Boston, Massachusetts, United States; Boston University, Boston, Massachusetts, United States; Boston University, Boston, Massachusetts, United States; Tufts Medical Center, Boston, Massachusetts, United States

## Abstract

Aging is a heterogeneous process that unfolds differently across individuals and biological systems. While single biological clocks provide valuable insights, they often fail to capture the complex and multidimensional nature of aging. In this study, we developed system-specific aging clocks using metabolomics data from the Integrative Longevity Omics study to better understand the heterogeneity of aging trajectories. Each clock was designed to estimate biological age within a distinct metabolic system, under the assumption that variability in system-specific function reflects unique aspects of the aging process. Our analyses revealed striking inter-individual variability: some participants consistently exhibited age acceleration across systems, others showed age deceleration, and many demonstrated mixed patterns depending on the system measured. To further explore this heterogeneity, we clustered participants into subgroups based on their system-specific aging profiles. We then examined associations between these subgroups and (1) the Nutrient Variety Index (NVI), a comprehensive metric summarizing dietary diversity across 19 nutrient groups, (2) cognitive performance, and (3) mortality risk. We found that several subgroups displayed significant associations with multiple NVIs, particularly those reflecting balanced intake of carbohydrates. These same subgroups also showed more favorable cognitive outcomes and reduced mortality risk, suggesting that consistent patterns of healthy aging may be linked to dietary diversity and nutritional balance. Conversely, other subgroups displayed discordant patterns of aging acceleration and were associated with poorer outcomes. These findings highlight that aging is not uniform but system-specific, and that metabolomic aging clocks offer a promising framework for uncovering distinct pathways shaping healthy aging.

